# Peptide Photoimmobilization by Thiol–ene Chemistry for Enhanced Neural Cell Adhesion

**DOI:** 10.1021/acsbiomaterials.5c00853

**Published:** 2025-10-07

**Authors:** Yu-Liang Tsai, Sotiria Moschopoulou-Triantafyllidou, Jiyao Yu, Sa’id Albarqawi, Tommaso Marchesi D‘Alvise, Lothar Veith, Rüdiger Berger, Christopher V. Synatschke

**Affiliations:** † Synthesis of Macromolecules, 28308 Max Planck Institute for Polymer Research, Ackermannweg 10, 55128 Mainz, Germany; ‡ Physics at Interfaces, Max Planck Institute for Polymer Research, Ackermannweg 10, 55128 Mainz, Germany

**Keywords:** surface modification, photopatterning, thiol–ene reaction, neural cell adhesion

## Abstract

Neurological diseases and neural injuries are prevalent but difficult to treat because of the complexity of the neural environment. To unravel this complexity, simple cell culture models are required that allow the study of individual aspects of the neural environment under defined conditions. In this work, we developed stable coatings of bioactive peptides via photoimmobilization through a thiol–ene reaction on glass substrates suitable for long-term culture of neural cells. The substrates were modified with thiol groups via chemical vapor deposition to obtain a homogeneous layer, followed by the immobilization of neural active peptides bearing vinyl groups. Subsequently, human neuroblastoma cells were shown to stably adhere to and grow on the modified substrates. The results establish a facile fabrication route for patternable and peptide-functionalized substrates for the culturing of neural cells without an additional antifouling treatment.

## Graphical abstract

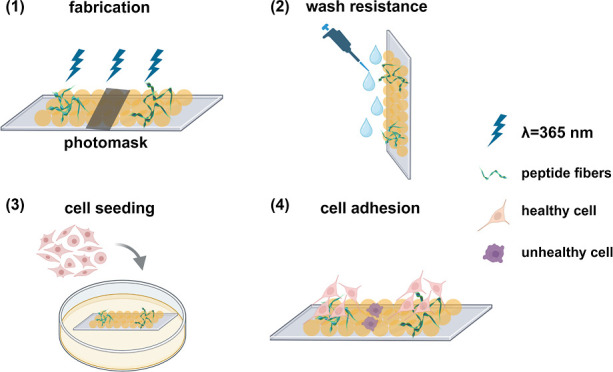

## Introduction

Protein and peptide immobilization on substrates through covalent bonds provides an effective way to introduce new functionalities to the substrate, such as adhesion, biocompatibility, antifouling, or biosensing.^1–5^ Additionally, having a solid support can improve the stability of the target proteins and peptides.^6^ With a proper orientation of the target molecules on substrates, it is possible to maximize the selectivity and reactivity by exposing the binding domain or the bioactive site in the solutions.^7^ To generate biomolecule-modified substrates, photopatterning holds the advantage of rapid fabrication, which is beneficial for high-throughput analysis in cell culture, e.g., when developing neural regeneration strategies. Among various photopatterning techniques, the photochemistry of olefins and thiols, which is commonly known as the thiol–ene reaction, has many advantages, such as regiospecificity, mild reaction conditions, absence of heavy metals, and high yield.^3,8–10^ Jonkheijm et al. demonstrated that thiol–ene photochemistry could form stable thioether bonds to immobilize proteins with high precision on glass slides in physiological buffers.^8^ Herein, we aim to combine the advantages of thiol–ene photochemistry and bioactive peptides to rapidly create a robust platform for neural cell adhesion. The bioactive peptide sequence, KIKIQIN, is derived from a self-assembling amyloid-like peptide consisting of amphiphilic 12-amino acid, termed an enhancing factor *C* (EF-*C*). The EF-*C* peptide and its derivatives were employed to enhance retroviral gene transfer^11^ and peripheral nerve repair.^12^ Previous work has established that the self-assembling structure plays an important role in the functionality, but little attention has been paid to the functionality of such materials on substrates. Herein, the EF-*C-*derived peptide sequence was selectively immobilized on substrates by thiol–ene photochemistry, as depicted in Figure 1, to build a robust neuronal cell culture platform.

**Figure 1:**
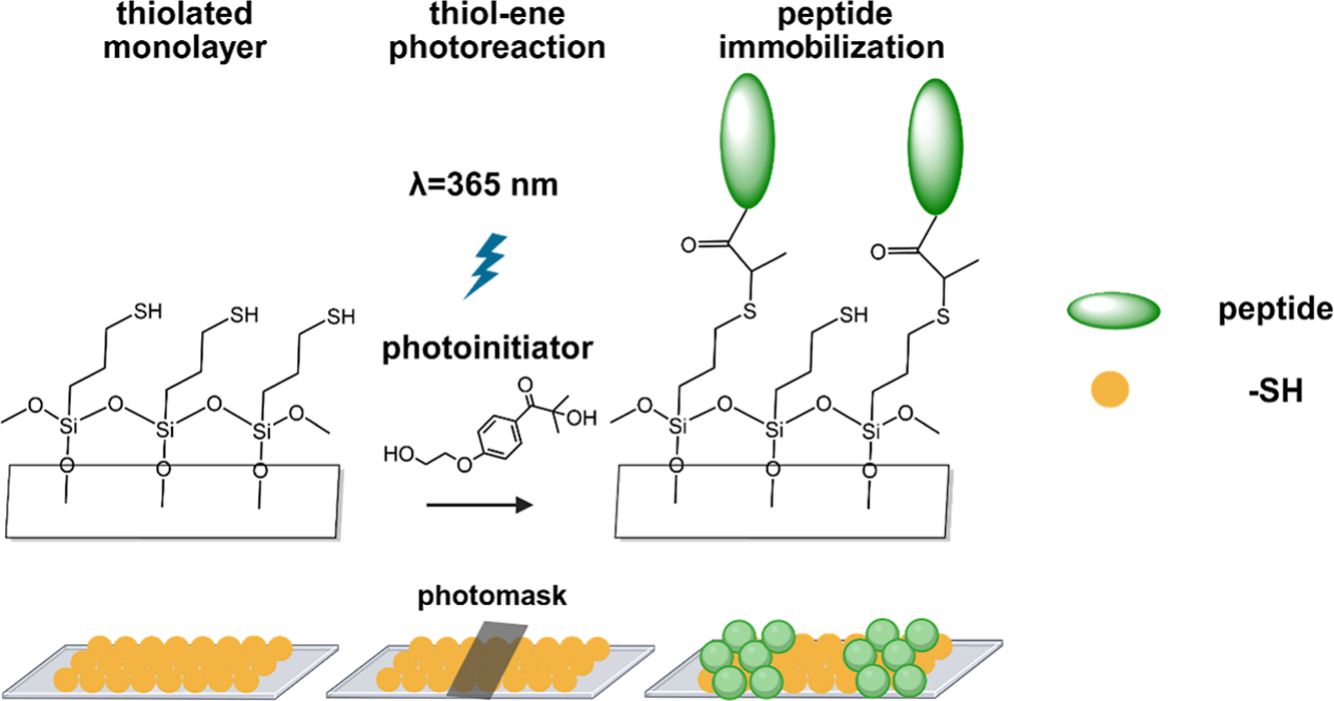
Chemical reaction scheme and conceptual design of the peptide surface modification. The fabrication process of peptide immobilization on the substrate started with chemical vapor deposition to generate a thiol layer on a glass substrate, followed by photon-activated thiol–ene reaction with acrylated peptides (sequence: Acr-KIKIQIN). (This image was created using ChemDraw and BioRender.)

Surface characteristics such as hydrophilicity, surface charges, surface topography, and surface energy can influence cellular behaviors such as achieving cell adhesion, cell alignment, cell migration, directing morphological changes, activating signaling pathways, and inducing differentiation.^4,5,13–17^ Cells respond to these features, provided by their surroundings at multiple scales from the macroscale down to the nanoscale.^18^ Neuronal cells, in particular, are responsive to physical cues that direct neuronal cell faith.^19–23^ For instance, Zhang et al. covalently patterned peptide sequences derived from laminin in a gradient manner on poly­(*D*, *l* -lactide-co-caprolactone) (PLCL) films. The micropatterns and peptide gradient on the PLCL films improved the directional growth of mouse Schwann cells.^24^ Tomba et al. reported that glial cells responded to the stiffness on different substrates, i.e., they favored cell adhesion and proliferation on stiffer fibronectin substrates but softer poly- *l* -lysine/laminin substrates.^22^ Vedaraman and colleagues reported that using two-photon lithography techniques to design periodic and anisometric patterned features can create a high-throughput platform for neurite alignment.^23^

Different methods for the fabrication of topographic features have been developed, including the use of peptide photopatterning and self-assembling peptides (SAP), allowing for the investigation of the neural behavior. For example, SAPs can provide biomimetic complex structures for neural cell attachment.^14,19^ Yang et al. fabricated an aligned nanofiber composed of fibrin and SAPs to stimulate the outgrowth of rat Schwann cells and the functional recovery in a peripheral nerve injury in rats.^25^ In addition, 3D printing is one technique to generate an aligned and functional network for neural tissue engineering.^26,27^ Indeed, photopatterning, SAPs, electrospun fibers, and 3D-printed constructs can create complex matrices that mimic neural cell niches. However, the abovementioned techniques alone cannot rapidly fabricate patterned substrates to direct the neuronal cell behavior.^15^

In this work, we aim to immobilize the self-assembling and bioactive peptide KIKIQIN, via thiol–ene photochemistry, and investigate its functionality on the modified surfaces. We characterize the physical and chemical properties of the acrylated KIKIQIN peptide and verify the potential of photoimmobilization of this bioactive sequence on substrates. Finally, cell adhesion assay of a human neuroblastoma cell (SH-SY5Y) is used to test the functionality of the photoimmobilized peptide to achieve a robust neuronal cell culture platform.

## Experimental Section

### Materials

*N,N*-Dimethylformamide (DMF), dichloromethane (DCM), 2-propanol, piperidine, and trifluoroacetic acid (TFA) were purchased from Carl Roth GmbH + Co. KG. Acetonitrile (ACN, HPLC grade), acetone, diethyl ether, and toluene were obtained from Honeywell. Fluorenylmethyloxycarbonyl (Fmoc)-protected amino acids, Fmoc-Asn (Trt) Wang resin, and ethyl cyano­(hydroxyimino) acetate (Oxyma Pure) were purchased from Novabiochem. α-Cyano-4-hydroxycinnamic acid (CHCA), *N,N*’-diisopropylcarbodiimide (DIC), *N,N*-diisopropylethylamine (DIPEA), dimethyl sulfoxide (DMSO), 2-hydroxy-4′-(2-hydroxyethoxy)-2-methylpropiophenone (Irgacure 2959), and triisopropylsilane (TIPS) were acquired from Sigma–Aldrich. Acrylic acid anhydride and (3-mercaptopropyl) trimethoxysilane (MPS) were bought from TCI Deutschland GmbH. Dithiothreitol was purchased from VWR. All chemicals were used without further purification unless otherwise noted. Ultrapure water was obtained by using a purification system (Milli-Q, Merck KGaA).

### Solid-Phase Peptide Synthesis and Peptide Acrylation

Peptide synthesis (KIKIQIN) was performed in an automated microwave peptide synthesizer (Liberty Blue, CEM Corporation) from the *C*- to *N*-terminus by the Fmoc-SPPS method using Fmoc-Asn (Trt) Wang resin with 100–200 mesh size. DMF was used as the main wash solvent, and DMF with 20% piperidine was the deprotection solvent. The amounts of materials and reagents were prepared according to the calculation suggested by the software of Liberty Blue. In brief, the Wang resin was swelled in DMF for 30 min prior to the synthesis. Routinely, a single amino acid coupling was applied unless otherwise stated. The procedure started from Fmoc removal by immersing the resin in the deprotection solvent and heating to 75 °C (155 W) for 15 s and 90 °C (30 W) for 50 s, followed by washing with DMF twice. Subsequently, amino acids were coupled using a 0.2 M solution of the respective amino acid in DMF with the addition of activator and activator base. The reaction was heated to 75 °C (170 W) for 15 s and 90 °C (30 W) for 110 s, followed by the final deprotection step and flushing with DMF. Acylation of peptide (Acr-KIKIQIN) was conducted on the resin with a stoichiometry of 1:2:4 (peptide: AA: DIPEA) in DMF in a peptide reactor (Carl Roth GmbH + Co. KG) by shaking for 4 h at room temperature. Afterward, resin beads were first rinsed with DCM and immersed in a cleavage cocktail (95% TFA, 2.5% Milli-Q water, and 2.5% TIPS) for 2 h, followed by precipitation in cold diethyl ether and centrifugation to obtain the crude peptide.

### Purification and Characterization of Acylated Peptide

The crude peptide was dissolved in a mixture of Milli-Q water and ACN and purified by reverse-phase liquid chromatography (RP-HPLC, Shimadzu) through a C18 column (Phenomenex Gemini, 5 μm, NX-C18, 110 Å, 150 × 30 mm) with a flow rate of 25 mL/min. The solvent gradient of a mixture of ACN/Milli-*Q* with 0.1% TFA started from 10 to 100% ACN. Fractions of samples were collected according to the retention time detected by an ultraviolet (UV) absorption detector at 214 nm. The collected samples were identified by matrix-assisted laser desorption/ionization time-of-flight mass spectrometry (MALDI-ToF MS) via a dried droplet method. Samples were mixed with a saturated solution (Milli-Q water/ACN = 1:1) of the matrix CHCA. The mass spectra were recorded on a rapifleX MALDI-ToF/ToF (Bruker) system. The purified samples were lyophilized and stored at −20 °C until further use.

### Substrate Thiolation

Thiolation of the substrate was conducted by vapor deposition based on a previous report.^28^ Glass slides (microscope slides, Epredia) for cell experiments and silicon wafers (*P*/Boron, ⟨100⟩, Si-Mat) for physicochemical analysis were cleaned by sonication in 2-propanol for 30 min. Afterward, the substrates were rinsed with Milli-Q water and dried with a N_2_ stream. A polydimethylsiloxane (PDMS) elastomer film was prepared by mixing a base elastomer and curing agent at a 10:1 weight ratio from Dow Corning’s Sylgard 184 elastomer kit following the manufacturer’s instructions. The mixture was thoroughly stirred, degassed under vacuum to remove air bubbles, and was poured into a clean Petri dish to achieve a film thickness of 3 mm. Finally, PDMS was cured by heating it at 60 °C for 2 h, resulting in a flexible, transparent elastomeric film. Afterward, the PDMS films were gently peeled from the dish and were treated with oxygen plasma (Femto low-pressure plasma system, Diener electronic GmbH, Germany) for 30 s at 200 W to enhance their reactivity and generate surface hydroxyl groups.^29^

Clean substrates and a Teflon bottle with an open vial containing 0.5 mL of MPS were placed in a desiccator. The desiccator was purged with N_2_ stream, sealed, and placed in a 90 °C oven for 1 h. Subsequently, the substrates were rinsed with toluene, followed by acetone, and then dried using a N_2_ stream.

### Peptide Photoimmobilization on Thiolated Substrates

Different concentrations (0.5, 0.1, and 0.05 mg/mL) of peptide powders were dissolved in a solvent mixture of Milli-Q water (90%) and ACN (10%). Photoinitiator, Irgacure 2959, was solubilized in the same solvent mixture with a concentration of 1 mg/mL. Peptide and photoinitiator solutions were mixed and applied to the thiolated substrates for 5 min of ultraviolet (UV) irradiation (1.6 mW/cm^2^). The peptide-immobilized substrates were washed with DMSO and Milli-Q water and then dried with a N_2_ stream prior to further experiments.

### X-Ray Photoelectron Spectroscopy

X-Ray photoelectron spectroscopy (XPS) was conducted using a Kratos Axis UltraDLD spectrometer (Kratos) with an Al Kα excitation source with a photon energy of 1486.6 eV. Each sample was measured at three different spots, and data were processed by the CasaXPS software and plotted by Origin.

### Time-of-Flight Secondary Ion Mass Spectrometry

Time-of-flight secondary ion mass spectrometry (ToF-SIMS) experiments were performed using a TOF.SIMS5 (NCS) instrument (IONTOF GmbH) with 30 keV of Bi_3_^+^ primary ions. Large-area images were acquired using 30 keV Bi_3_ primary ions rastering a total area of 7 × 15 mm^2^ in spectrometry mode at a current of 0.18 pA and a cycle time of 150 μs (mass range up to 1800 *m*/*z*), leading to imaging data sets with pixel sizes of 3.3 × 3.3 μm^2^. Mass calibration was facilitated by using ubiquitous aliphatic hydrocarbon species.

### Contact Angle Measurement

The wettability of the substrates was analyzed by measuring static contact angles by using the sessile drop method (DataPhysics OCA 35 contact angle goniometer). Triplicate measurements were performed by depositing 10 μL of Milli-Q water on three different spots per substrate. The data were processed within DataPhysics and plotted using Prism.

### Surface Zeta Potential

Zeta potential measurements were conducted using a SurPASS electrokinetic analyzer (Anton Paar GmbH) to evaluate the surface charge properties of the thiolated and peptide-modified substrates under physiological conditions (pH 7.4). A 1 mM KCl solution served as the electrolyte, while 0.1 M HCl and 0.1 M NaOH were used to adjust the pH. Samples were mounted in a clamping cell, with the channel height adjusted to approximately 100 μm. Electrokinetic flow was induced by linearly ramping the differential pressure from 0 to 300 mbar, and the streaming potential was recorded as a function of the applied pressure. Each sample was measured in triplicate, and the data were plotted using Prism.

### Scanning Force Microscopy

The surface morphology of the thiolated and the peptide-modified substrates was measured by scanning force microscopy (SFM) (Dimension Icon, Nanoscope 5 controller, Software 9.7r1sr8) operated in soft tapping mode at a scan speed of around 1 Hz with 512 pixels per line and 512 lines. We used cantilevers with a nominal resonance frequency of 300 kHz and a nominal spring constant of 26 N/m (OTESPA, OPUS made by μ mash). All images were plane-corrected by an offset and a tilt (first-order polynomial). The height of the fibers was determined from surface profiles.

### Photoinduced Force Microscopy

Nanoinfrared (IR) spectroscopic microscopy (VistaScope, MolecularVista) in the photoinduced force microscopy (PiFM) mode^30,31^ was used to map the secondary structure of the immobilized peptide. Surface topography was recorded by exciting the second eigenmode of the cantilever resonance frequency. For topography imaging, a constant vibrational amplitude was maintained using an electronic feedback circuit. The forces induced by the IR light (Quantum Cascade Laser, Block Engineering) were recorded at the first eigenmode of the cantilever resonance frequency. The incoming focused IR light was modulated at the difference frequency between the first and second eigenmodes. This difference frequency was fine-tuned to the maximum response vibrational amplitude of the cantilever at the first eigenmode, while the tip was engaged with the surface. We selected a wavenumber of 1690 cm^–1^ and another one of 1750 cm^–1^ as nonspecific references. We applied a Gaussian filter to both nano-IR images, averaged 5 adjacent pixels, and extracted the line profiles of the IR response across a fiber structure.

### Cell Culture of Human Neuroblastoma Cells (SH-SY5Y)

SH-SY5Y cells (ATCC- CRL-2266) were cultivated at 37 °C, 95% humidity, and 5% CO_2_ in Dulbecco’s modified Eagle’s medium/Ham’s *F*-12 Nutrient Mixture (*F*-12) (Thermo Fisher Scientific) with additional 10% fetal bovine serum, 1% GlutaMax (Thermo Fisher Scientific), and 1% penicillin–streptomycin (PS) (Sigma–Aldrich) to study cell adhesion on thiolated and peptide-modified glass slides.

### Cell Adhesion Assay

Prior to cell seeding, all substrates were washed with DMSO, Milli-Q water, and DPBS (phosphate-buffered saline without calcium chloride and magnesium chloride, Thermo Fisher Scientific). Subsequently, the liquid residual on substrates was removed using a vacuum aspiration system (VACUBOY, INTEGRA Biosciences). All substrates were placed in a 6-well plate. Then, cells were seeded on a bare glass (blank), thiolated glass, peptide drop-cast glass (0.1 mg/mL), different concentrations of peptide-immobilized substrates (0.05, 0.1, and 0.5 mg/mL), and the peptide-photopatterned substrate (0.1 mg/mL) at a density of 100,000 cells/cm^2^ for 72 h. The culture medium was changed every 24 h before observation. For better visualization and quantification, cell nuclei were stained with blue fluorophore (NucBlue Live ReadyProbes Reagent), and cell membranes were stained with red fluorophore (Invitrogen CellMask DeepRed Plasma Membrane Stain) according to the manufacturer’s protocol (Thermo Fisher Scientific). For immunostaining, cells were rinsed with PBS, fixed with 4% (v/v) paraformaldehyde (PFA, Sigma–Aldrich) for 10 min, permeabilized with 0.5% (v/v) Triton *X*-100 (Merck KGaA) for 5 min, and blocked with 4% bovine serum albumin (BSA, Roche) in PBS for 5 min. The primary antibody synaptophysin (SYN, rabbit *anti*-SYN, Proteintech) was diluted in PBS (1:1000) and added to the samples at 4 °C for 24 h. After rinsing with PBS, the secondary antibody labeled with Alexa 488 (donkey antirabbit IgG Alexa Fluor Plus 488, Invitrogen, Thermo Fisher Scientific) in PBS (1:1000) was added to samples at room temperature for 4 h before imaging. The images were taken using a BZ-X800 microscope (Keyence) utilizing a Plan Fluorite 20X LD PH objective lens. The built-in “Navigation” and “Stitching” functions of the microscope were used to visualize a larger area. The Navigation function starts recording images from a selected image and spirally outward, while the Stitching function records 84 images within a selected area. Image files were processed with NIH ImageJ software, and the “analyze particles” function was used to measure the total number of cells in a determined area.^32^ Statistical analysis was performed using the one-way ANOVA test and plotted using Prism.

## Results and Discussion

### Chemical Characterization of Peptide-Modified Substrates

To achieve peptide photoimmobilization on substrates by thiol–ene photochemistry, we chose KIKIQIN, as this peptide has previously been shown to facilitate neuronal adhesion on glass substrates.^12^ The peptide was synthesized using Solid-Phase Peptide Synthesis, followed by on-resin acrylation at the *N*-terminus, and the chemical structure of Acr-KIKIQIN is provided in Figure S1. The resulting Acr-KIKIQIN was cleaved off the resin, purified by RP-HPLC, and confirmed by MALDI-ToF MS (Figure S1).

Next, glass and silicon substrates were modified with MPS via vapor deposition to introduce thiol groups on the surface, followed by a photoinitiated thiol–ene reaction with Acr-KIKIQIN. A simple strip-patterned photomask, depicted in Figure 2a, was used for photopatterning of Acr-KIKIQIN on the thiolated substrates. The chemical characterization of the modified substrates was carried out by XPS, where the differences between thiolated and peptide-modified substrates could be easily distinguished in the obtained spectra. In the *C* 1s spectra of the thiolated substrate shown in Figure 2b, the brown dashed line was attributed to the C–C bonds at 284.9 eV, the blue dotted line was assigned to the C–O bonds at 286.8 eV, and the green dashed line was correlated to the C=O bonds at 289. 2 eV. In contrast, the *C* 1s spectra of the peptide-modified substrates, shown in Figure 2c, showed additional signals corresponding to the C–N bonds at 286.2 eV (dark yellow dashed line). There are significant differences in these two *C* 1s spectra in the intensity of the signal at 288–289 eV attributed to the C=O bonds and a subtle difference at 286.2 eV assigned to the C–N and C–O bonds. The profound signals from C=O bonds and C–N bonds are typical signals from peptide bonds, which served as a strong indication of peptide immobilization on the substrate. The elemental composition analysis was performed using the deconvoluted spectra and is summarized in Table 1.

**Figure 2:**
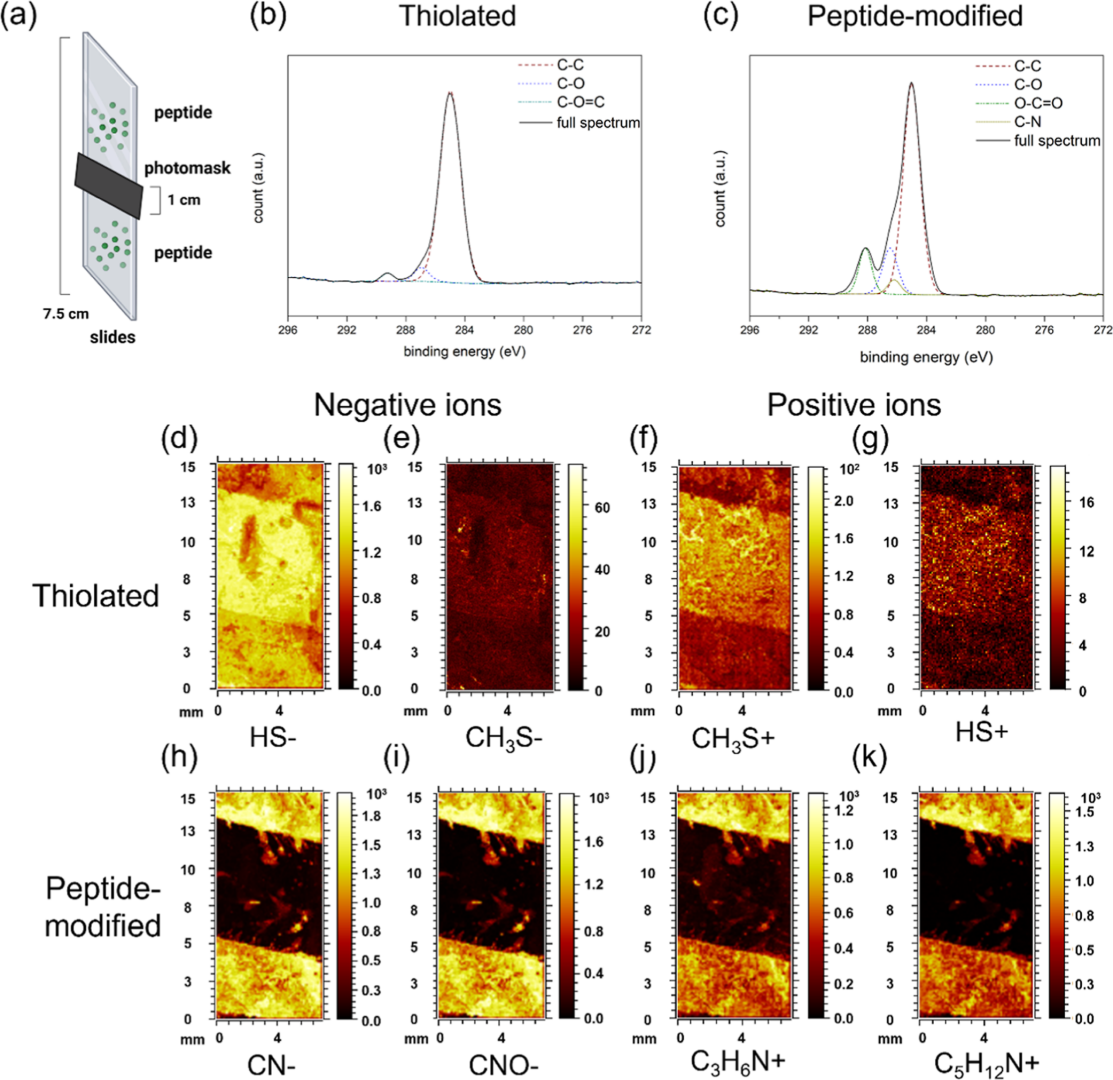
Surface chemical characterization of thiolated and peptide-modified substrates. (a) Scheme of peptide photopatterning on the thiolated glass slides. (This image was created using BioRender.) (b) High-resolution narrow scan of *C* 1s of the thiolated side of the substrate and the deconvoluted spectra for the probable chemical species. (c) High-resolution narrow scan of *C* 1s of the peptide-modified side of the substrate and the deconvoluted spectra for the probable chemical species. (d–g) Representative ToF-SIMS chemical maps of the thiolated substrates, with HS^–^, CH_3_S^–^, CH_3_S^+^, and HS^+^. (h–k) Representative ToF-SIMS chemical maps of the peptide-modified substrates (CN^–^, CNO^–^, C_3_H_6_N^+^, and C_5_H_12_N^+^).

**Table 1:** Elemental Composition Analysis of Thiolated and Peptide-Modified Silicon Wafers Based on Their XPS Spectra

	element content (wt%)			
samples	*C* 1s	*O* 1s	*N* 1s	*S* 2p
thiolated	36.7	40.7	0.9	21.7
peptide	49.7	32.9	6.7	10.7

The same synthetic route can also be applied to modify the PDMS films. In Figure S2­(a,c), there are negligible differences in the high-resolution *C* 1s spectra between thiolated and peptide-modified substrates due to strong contribution from the PDMS film. However, the signal at 400–401 eV in *N* 1s spectra attributed to nitrogen in the interstitial position within the lattice was only present in the peptide-modified substrate,^33^ as shown in Figure S2­(b,d), indicating successful modification.

Next, ToF-SIMS was applied to investigate the chemical composition of the substrate and to provide further evidence of successful peptide modification. Because both MPS and peptides contain a significant amount of aliphatic groups, peptide-associated signals were mainly originating from aliphatic amines (C–N signals), while thiol-associated signals were originating from fragments containing sulfur in combination with carbon and hydrogen. In Figures 2a–h and S3­(a,b), ToF-SIMS images from the photopatterned peptide-modified substrates in positive and negative ions are presented. A semiquantitative analysis of the relevant ionic signals is presented in Table 2. As expected, nonilluminated regions primarily showed signals that originate from the thiol groups of MPS, confirming that the coupling of peptide in nonilluminated areas is negligible. In contrast, illuminated areas showed strong signals (10-fold higher intensity compared to nonilluminated areas) related to the peptide fragment, confirming the successful coupling and photopatterning of the substrate. Additionally, the signal of [M + H]^+^ = 924.5 *m*/*z* in Figure S4, which matched the molecular weight of Acr-KIKIQIN, was also captured by ToF-SIMS, indicating the successful peptide modification on the substrate.

**Table 2:**
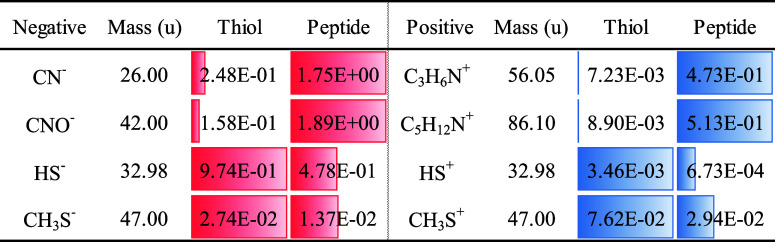
Semiquantitative Analysis of Relevant Ionic Signals Detected by ToF-SIMS (Unit: Area Normalized by Total Shots)^a^

a The color bars indicate the relative intensity of the captured ions on the thiolated side and peptide-modified side of the substrate.

In short, both XPS spectra and ToF-SIMS suggested successful peptide immobilization on the substrate, and the ToF-SIMS images demonstrated successful photopatterning of Acr-KIKIQIN on thiolated substrates.

### Physical Characterization of Peptide-Modified Substrates

The functionalization with peptides should lead to a change in the surface properties of the samples, ideally providing a beneficial surface for the cells to attach. Next, we characterized the surface properties of the modified substrates in more detail and compared them to nonfunctionalized and thiolated substrates. Wetting analysis was tested by a contact angle goniometer and determined to be 51° ± 1°, 52° ± 2°, 61° ± 2°, 62° ± 3°, and 68° ± 3° for the blank, the thiolated, and (0.05 mg/mL, 0.1 mg/mL, and 0.5 mg/mL) peptide-modified substrates, as shown in Figure 3a. The increase in hydrophobicity with increasing peptide concentration was expected, as more peptides with aliphatic side chains were immobilized on the substrate.

**Figure 3:**
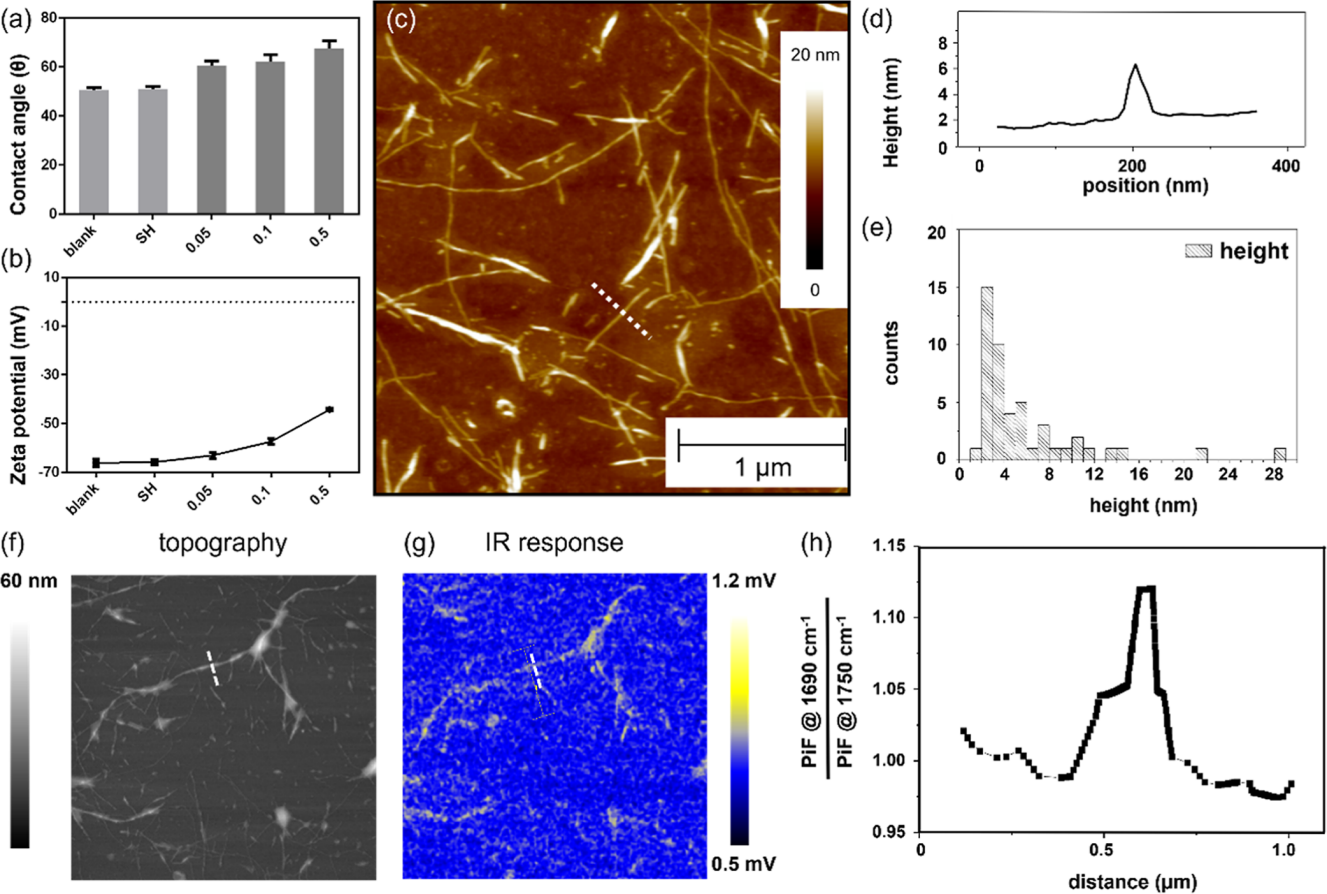
Physical characterization of the peptide-modified substrates. (a) Contact angle measurement of different substrates. Blank was bare glass (51° ± 1°), SH represented the thiolated glasses (52° ± 2°), and 0.05 mg/mL (61° ± 2°), 0.1 mg/mL (62° ± 3°), and 0.5 mg/mL (68° ± 3°) indicate the peptide concentrations (mg/mL) used for coating. (b) Surface zeta potential of different substrates. Blank was bare glass (−66.11 ± 1.58 mV), SH represented the thiolated glasses (−65.78 ± 0.72 mV), and 0.05 mg/mL (−62.98 ± 1.28 mV), 0.1 mg/mL (−57.31 ± 1.14), and 0.5 mg/mL (−44.17 ± 0.60) indicate the peptide concentrations (mg/mL) used for coating. (c) Representative SFM topography images of peptide-modified substrates (peptide concentration: 0.2 mg/mL). (d) Topography profile across a peptide nanofiber indicated by the white dotted line. We took the full width at half-maximum as the representative value for the height of a nanofiber. (e) Statistical analysis of the height of peptide nanofibers on the peptide-modified substrates. (f) Representative topography image of peptide-modified substrates (peptide concentration: 0.2 mg/mL). (g) Corresponding nano-IR map at 1690 cm^–1^. (h) Normalized intensity of the nano-IR signals at 1690 cm^–1^ and 1750 cm^–1^ across a selected peptide nanofiber indicated by the white dashed line in (f and g).

In surface zeta potential measurements, the modification of the substrate with the peptide can be observed as in Figure 3b. While there was no change in the surface zeta potential at pH = 7.4 when modifying the glass substrate with MPS (−66 mV for both glass and thiolated substrates), with increasing peptide concentration, an increase in the surface zeta potential from −63 mV for the lowest tested peptide concentration up to −44 mV for the highest tested peptide concentration was observed. In Figure S5, the increase in surface zeta potential at pH = 7.4, when modifying different peptide concentrations on PDMS substrates, verifies the successful modification and demonstrates the versatility of the approach toward polymeric substrates.^34,35^ The increase in the surface zeta potential could be attributed to multiple amine groups on lysine in the peptide sequence.

We further analyzed the surface morphology of the thiol- and peptide-modified Si substrate surfaces by SFM (Figures 3c–e and S6). Surprisingly, we found fiber-like structures on the surface, which had a height up to 30 nm and a width up to 100 nm. A statistical analysis of several images and 46 fibers revealed that most fibers had a height between 2 and 3 nm. The length of the fibers is in a range of hundreds of nanometers to several micrometers, indicating multiple hierarchies of the assembly. Based on the available data, we cannot distinguish if a seeded growth mechanism is taking place or if preformed fibers were attached. In order to further analyze the secondary structure of these nanofibers, PiFM was implemented to investigate the nano-IR response. First, we selected a wavenumber of 1690 cm^–1^, which represents the secondary structure of β-sheet (Figure 3f,g).^36,37^ The same area was imaged again at a wavenumber of 1750 cm^–1^ as a nonspecific reference. We analyzed a nano-IR line profile across a nanofiber, as sketched by the dashed line in Figure 3f,g. We plotted the nano-IR response which was normalized by the nonspecific response recorded at 1750 cm^–1^. The higher nano-IR signal at 1690 cm^–1^ at the position of the fiber indicates that these fibers had β-sheet and behave similarly as an amyloid-like peptide on the surface Figure 3h.

Many factors, such as surface charge, surface topography, chemical templating, and the presence of molecules, play a role in surface self-assembly.^38–41^ Although the exact surface-mediated self-assembly mechanism is unclear, we speculate that Arc-KIKIQIN was first immobilized on the thiolated layer as a nucleation site, followed by supramolecular peptide assembly, which is similar to previous works, i.e., where peptides assembled on highly oriented pyrolytic graphites,^42^ *N*-hydroxysuccinimide-modified glasses,^43^ maleimide-modified poly­(acrylic acid) substrates,^44^ and azide-modified wafers.^45^

### Neural Cell Adhesion

In previous studies on peptides of similar sequences to KIKIQIN, the so-called EF-C peptide and its derivatives were found to have biological activity as they improved retroviral gene transfer^11^ and enabled peripheral nerve repair.^12^ Drop casting was used to prepare peptide-coated substrates for in vitro tests as part of these biological assays. However, drop casting has several drawbacks, such as drying effects leading to inhomogeneous peptide concentrations and insufficient long-term stability when immersing the coating in buffers or cell culture medium. Therefore, substrates that were photocoupled with Acr-KIKIQIN were tested for their ability to provide a long-term and stable neural cell culture platform. Cell adhesion of a neuroblastoma cell line, SH-SY5Y, was quantified by the number of cells adhering to the substrates in two separate individual experiments, and 10 random images were analyzed from each experiment. For better visualization and to facilitate cell counting, cell nuclei were stained with NucBlue to provide a stronger contrast. In Figure 4a, a regular glass slide, the thiolated substrate, as well as a drop-cast sample, which underwent the same washing step, showed a low number of adhered cells, while a clear increase in attached cells was observed with the increasing peptide concentration, demonstrating that the cell-adhesive properties of KIKIQIN remain after covalent attachment to the substrate. Furthermore, the peptide-modified substrates showed a highly homogeneous coverage with cells over the whole substrate (Figure S7). However, the number of adherent cells did not correlate linearly with the concentration of the peptide modification. We speculated that this was due to the capacity of cell adhesion receptor and interface functionality.^46^

**Figure 4:**
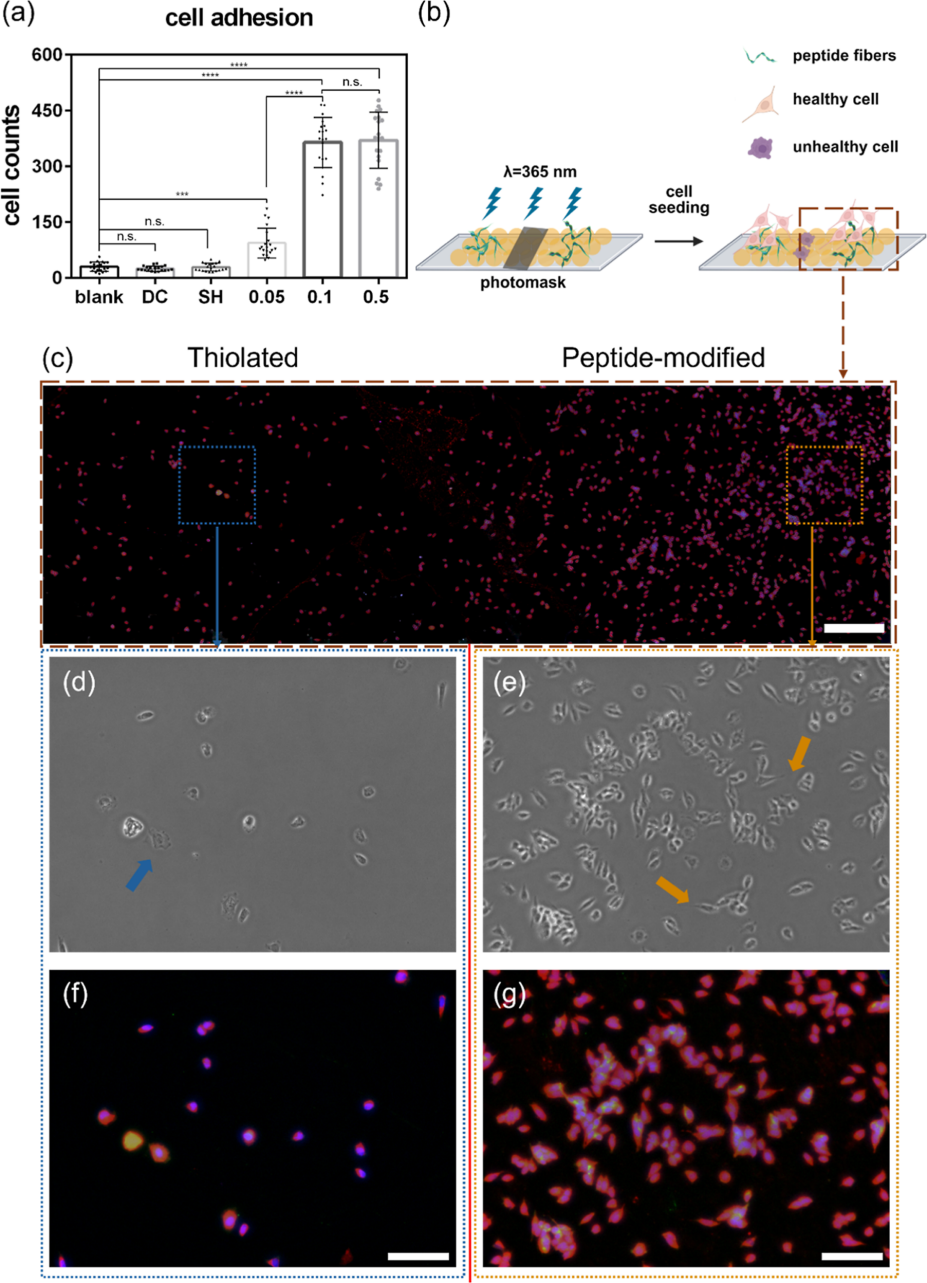
SH-SY5Y cell line in vitro assay. (a) Cell adhesion assay showed that the regular glass (blank), peptide drop-casted glass (DC), and thiolated glass (SH) had few cells adhered to the substrate, and the number of cells increased as the concentration of peptide modification increased (**p ≤ 0.01; ****p ≤ 0.0001, ns = not significant, *N* = 2, *n* = 20, one-way ANOVA test). (b) Conceptual scheme of peptide photopatterning for modulating cell adhesion (This image was created using BioRender.) (c) Overview image (stitched) of the patterned substrate with the thiolated side (left) and peptide-modified side (0.1 mg/mL) (right), with a scale bar of 600 μm. (d) Brightfield image of the zoomed-in region showing few and rounded (blue arrow) cells on the thiolated side of the substrate. (e) Brightfield image of the zoomed-in region showing many spreading cells (orange arrow) on the peptide-modified side of the substrate. (f) Fluorescent images of the zoomed-in region on the thiolated side of the substrate. (g) Fluorescent images of the zoomed-in region on the peptide-modified side of the substrate. Fluorescent images were acquired from stained samples, with nuclei in blue, cell membrane in red, and synaptophysin expression in green (scale bar = 100 μm).

In Figure 4b, a conceptual scheme demonstrates the cell adhesion after seeding on the photopatterned substrate. In Figure 4c, a stitched overview image shows a significantly larger number of adherent cells on the peptide-modified part (0.1 mg/mL) compared to the thiolated part of the substrate. Furthermore, clear differences in cell morphology are visible in the enlarged images, as shown in Figure 4d,e. While cells growing on the thiolated side of the substrate have a round shape, the cells growing on the peptide-modified side of the substrate are seen spreading and adhering to the substrate. Figure 4f,g shows the fluorescent images of the enlarged images. In Figure 4g, green fluorescent signals were observed from the Alexa 488 dye, which was from the secondary antibody against the expression of a neuronal marker, synaptophysin,^47^ whereas there was no green fluorophore in Figure 4f. In short, the thiolated area showed fewer cells adhering and with a rounded morphology, indicating that the unhealthy cells would detach or undergo apoptosis during prolonged cell culture. In contrast, the peptide-modified substrates showed a large number of healthy, spreading cells, demonstrating the suitability of the Acr-KIKIQIN peptide as a coating for long-term neuronal cell culture.

## Conclusions

An easily fabricated and robust platform for neural cell culture was developed that can accelerate the development of drug screening for neural-related diseases and neural tissue engineering. Here, a neuroactive peptide sequence was covalently attached to substrates via a phototriggered thiol–ene reaction, to provide robust coatings that promote cell adhesion. Surface chemical analysis showed that peptides could be selectively patterned on the substrate. Interestingly, SFM measurements showed that the immobilized peptides formed fibrous structures with dimensions of nanometers in height and micrometers in length, but the underlying mechanism for this behavior remains to be explored. Finally, superior cell adhesion of SH-SY5Y was demonstrated on the peptide-modified substrates, where increasing amounts of immobilized peptides showed an increase in the number of cells attaching to the surface when compared to nonpeptide-coated controls. Through photopatterning, our method allows us to structure the substrate and direct where cell adhesion can occur. This approach has the potential to create a robust and selective neural cell culture platform.

## Supplementary Material



## Notes

HPLC data on Acr-KIKIQIN for purity analysis; XPS, ToF-SIMS, zeta potential, and SFM for characterizing coated substrates; and overview images of SH-SY5Y cell adhesion on various substrates (PDF)
