# Supplementary material for: Peptide Photoimmobilization by Thiol–ene Chemistry for Enhanced Neural Cell Adhesion
Source: ACS Biomater Sci Eng. 2025 Oct 7;11(11):6844–53. doi: 10.1021/acsbiomaterials.5c00853 (PMC12606558; doi:10.1021/acsbiomaterials.5c00853)
Supplement: Supplementary file 1 [file ab5c00853_si_001.pdf]

Supporting information

Peptide photoimmobilization by thiol-ene chemistry for enhanced neural cell adhesion

Yu-Liang Tsai[1], Sotiria Moschopoulou-Triantafyllidou[1], Jiyao Yu[1], Sa'id Albarqawi[2], Tommaso Marchesi D'Alvise[1], Lothar Veith[1], Rüdiger Berger[2], Christopher V. Synatschke[1,*]

1. Max Planck Institute for Polymer Research, Synthesis of Macromolecules, Ackermannweg 10, 55128 Mainz, Germany
2. Max Planck Institute for Polymer Research, Physics at Interfaces, Ackermannweg 10, 55128 Mainz, Germany

* Corresponding Authors

E-mail: synatschke@mpip-mainz.mpg.de

Table of Contents

Figure S1. Chemical structure and purity analysis of acrylated peptide, Acr-KIKIQIN.

Figure S2. XPS of different PDMS substrate.

Figure S3. ToF-SIMS image of chemical 2D scans of the photopatterned peptide-modified substrates.

Figure S4. ToF-SIMS of the peptide modified substrate.

Figure S5. Surface zeta potential of different PDMS substrates.

Figure S6. SFM images of peptide different substrates at three different spots.

Figure S7. Overall images of SH-SY5Y cell adhesion on different substrates.

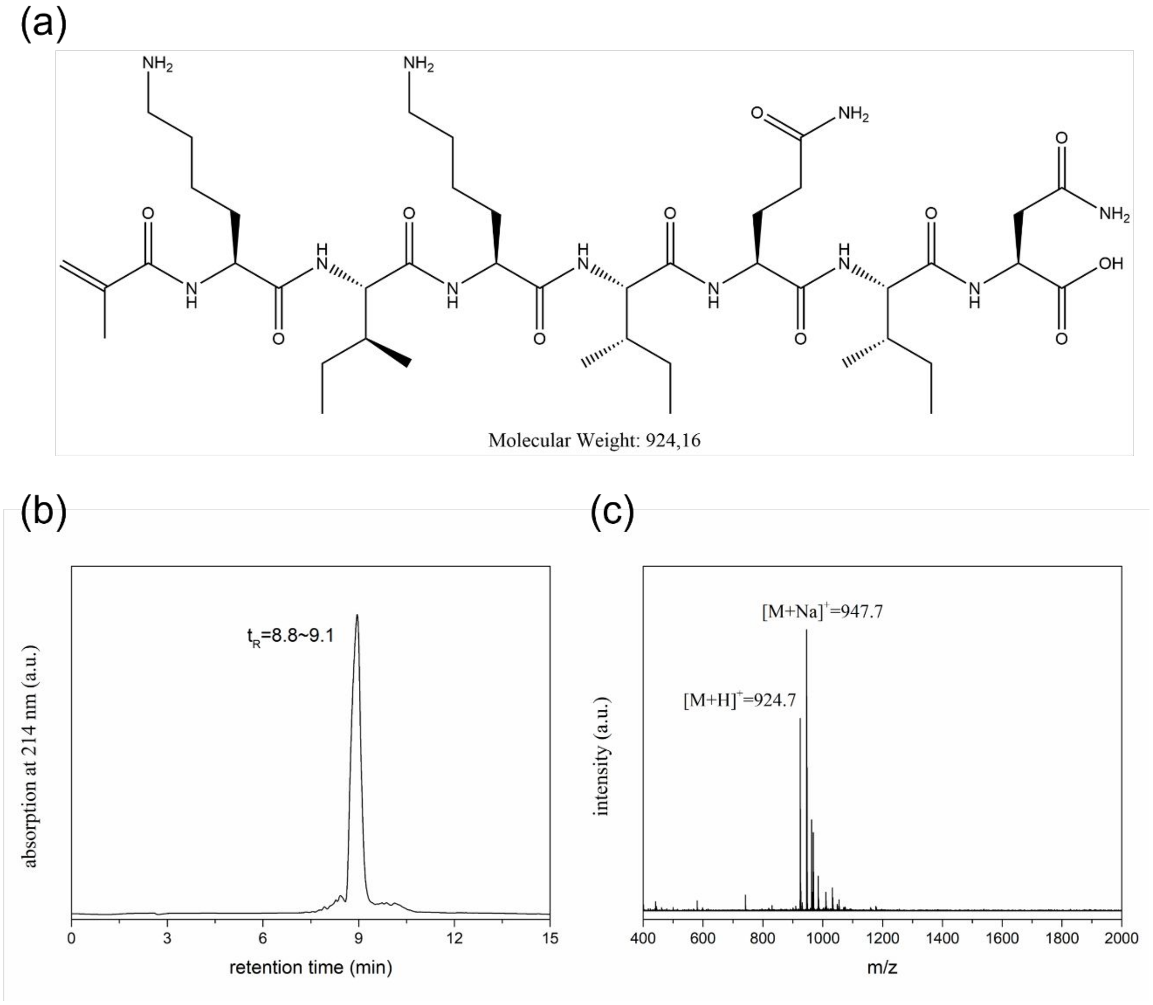

Figure S1. Chemical structure and purity analysis of acrylated peptide, Acr-KIKIQIN. (a) Chemical structure of Acr-KIKIQIN. (b) HPLC trace of the acrylated peptide in an acidic condition with a retention time (t_R) = 8.8– 9.1 min (purity > 95%). (c) MALDI-ToF-MS of Acr-KIKIQIN showing expected product signals assigned to $[M+H]^+$ =924.7 m/z and $[M+Na]^+$ = 947.7 were detected (theoretical MW = 924.2).

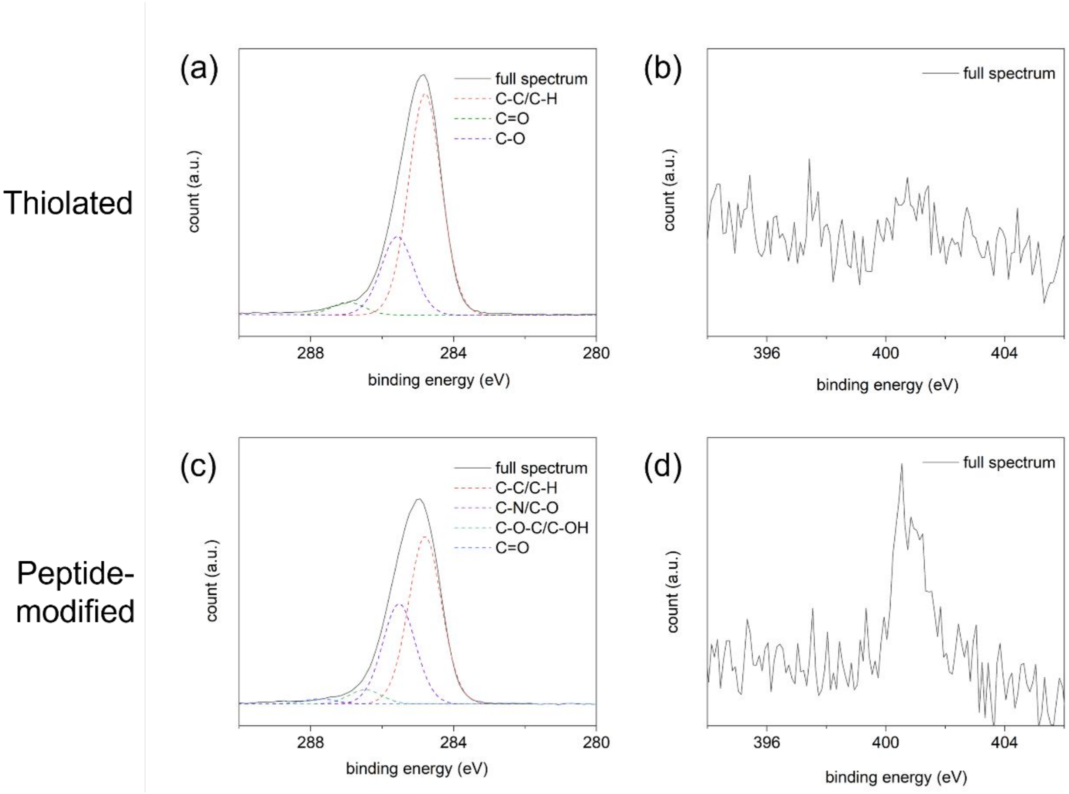

Figure S2. XPS of different PDMS substrates. (a) C1s spectrum of the thiolated PDMS film. (b) N1s spectrum of the thiolated PDMS film (c) C1s spectrum of the peptide-modified PDMS film. (d) N1s spectrum of the peptide-modified PDMS film.

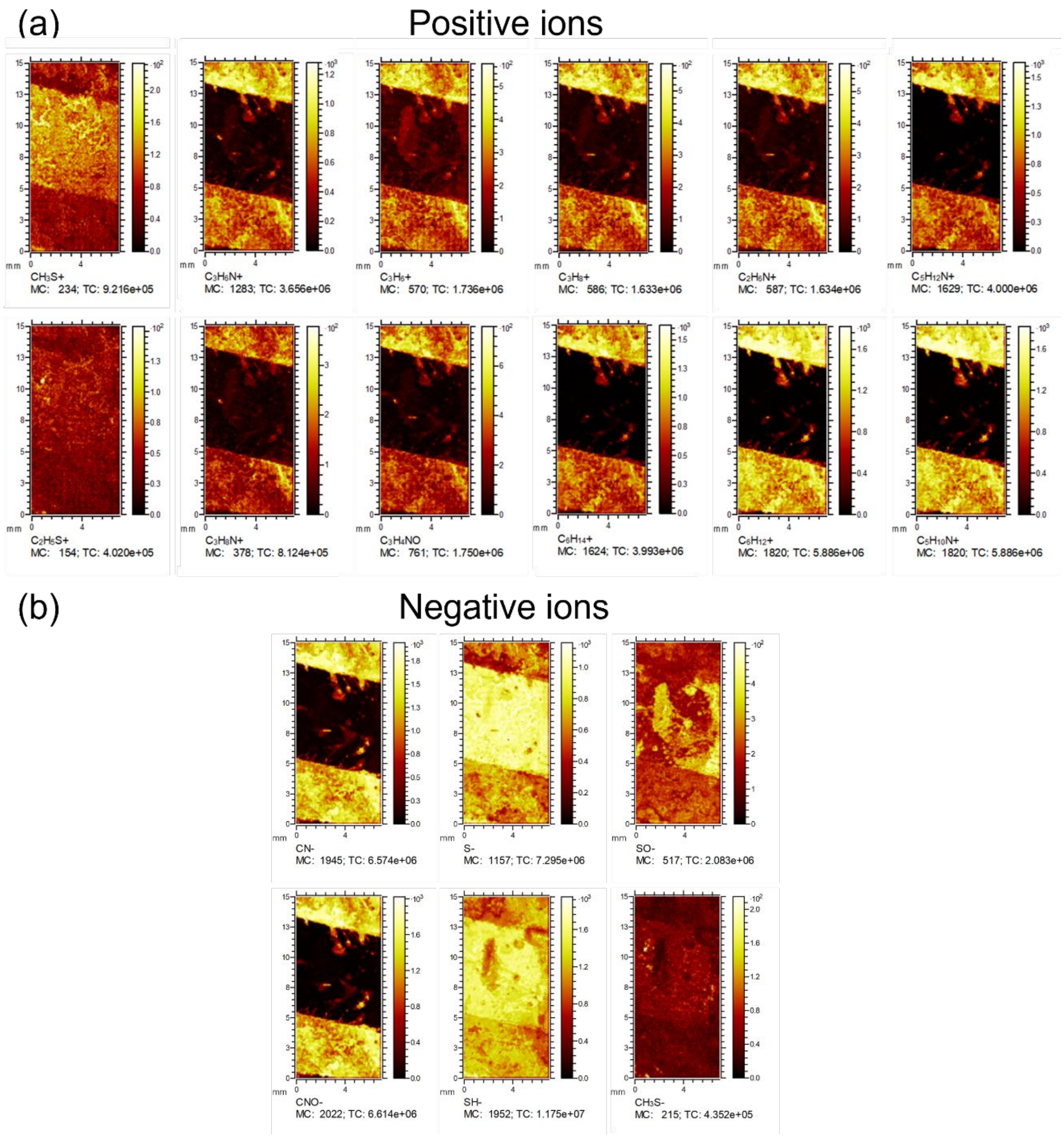

Figure S3. ToF-SIMS images of the photopatterned peptide-modified substrates. (a) Positive ions of ToF-SIMS images, including (CH_3S^+, $C_3H_6N^+$, $C_3H_6^+$, $C_3H_8^+$, $C_2H_6N^+$, $C_5H_{12}N^+$, $C_2H_5S^+$, $C_3H_8N^+$, C_3H_4NO, $C_6H_{14}^+$, $C_6H_{12}^+$, and $C_5H_{10}N^+$). (b) Negative ions of ToF-SIMS images, including (CN^-, S^-, SO^-, CNO^-, SH^-, and CH_3S^-). MC stands for maxium counts (per pixel) and TC represents total counts (in image).

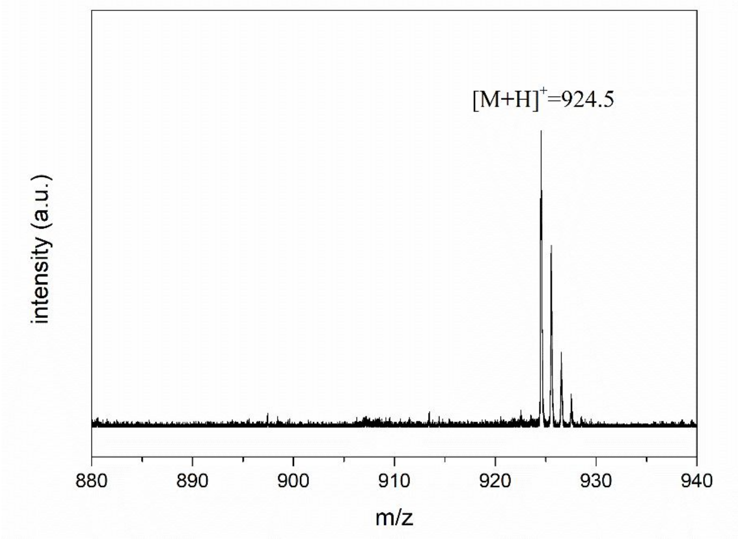

Figure S4. ToF-SIMS of the peptide modified substrate. $[M+H]^+$=924.5 m/z was detected (theoretical MW = 924.2) on the peptide modified substrate.

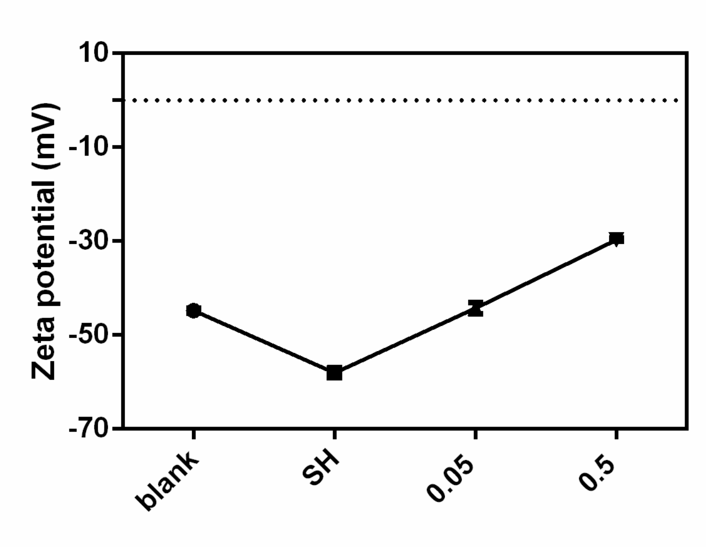

Figure S5. Surface zeta potential of different PDMS substrates. Blank was bare PDMS (-44.78 ± 0.51 mV), SH represented the thiolated PDMS (-58.01 ± 0.34 mV), and 0.05 mg/mL (-44.26 ± 1.24 mV), and 0.5 mg/mL (-29.70 ± 0.34) indicates the peptide concentrations (mg/mL) used for coating.

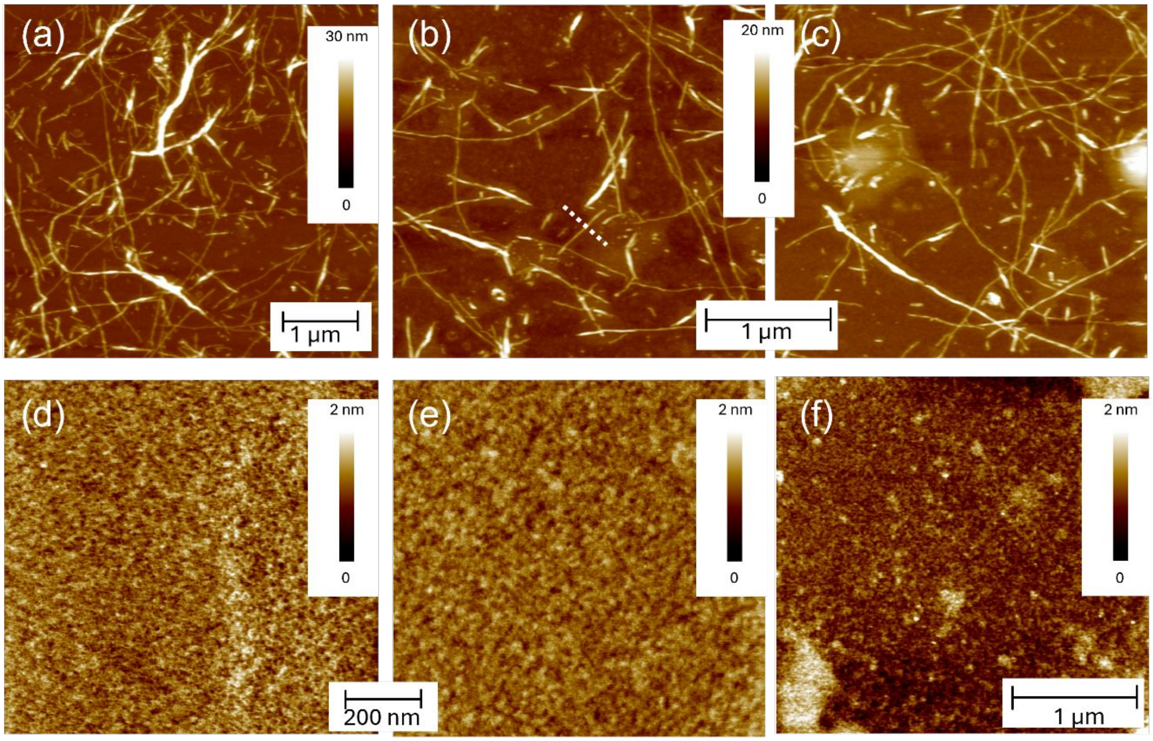

Figure S6. SFM images of photopatterned peptide-modified substrates at three different spots. (a, b, c) Peptide nanofibers are visible on the peptide-modified substrates in the SFM images. (d, e, f). Smooth surfaces without defined nanostructure are visible on the thiolated substrates in the SFM images.

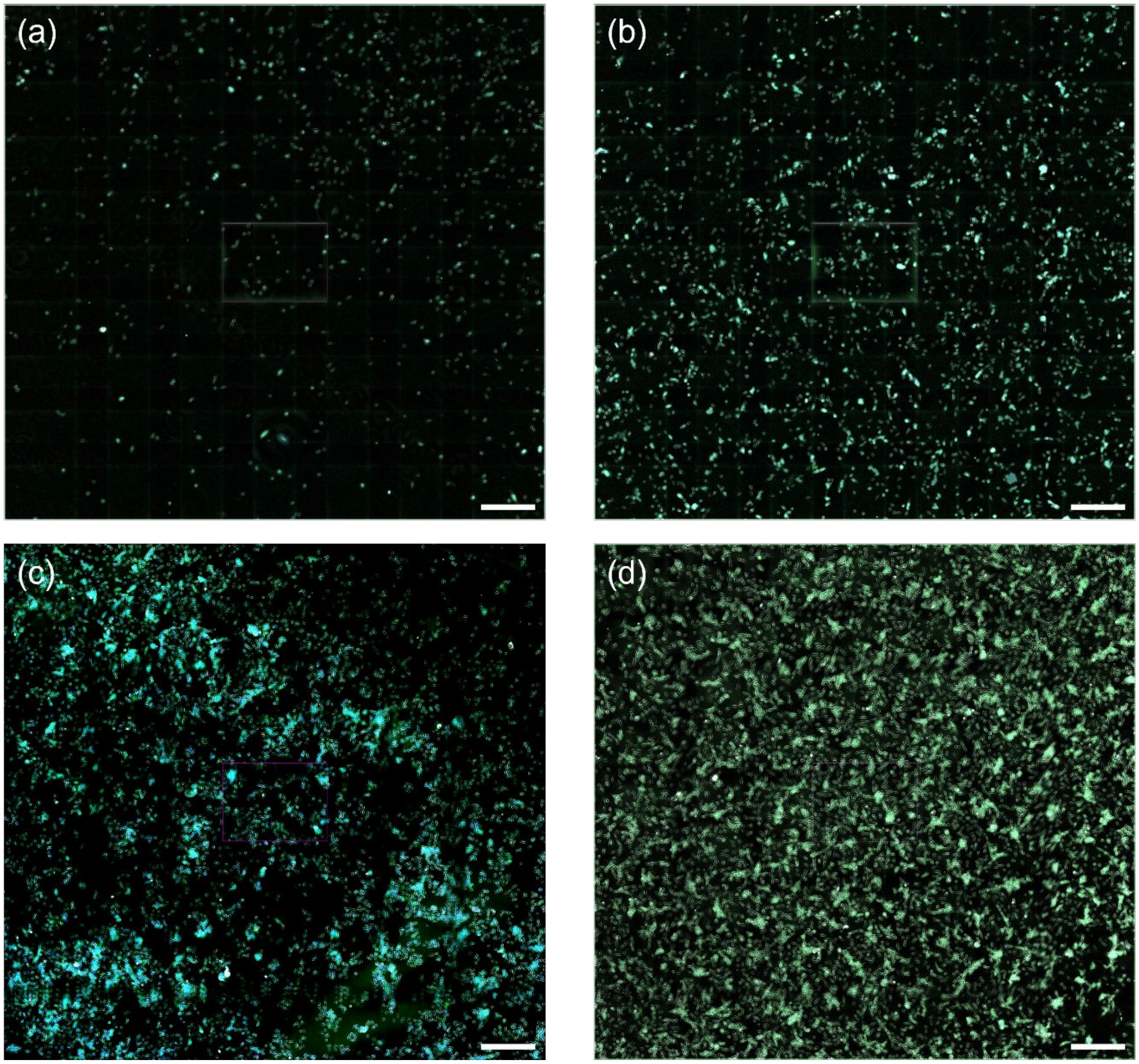

Figure S7. Large scale, stitched images of SH-SY5Y cell adhesion on different substrates. (a) Cell adhesion on the thiolated substrates. (b) Cell adhesion on the peptide (0.05 mg/mL) modified substrate. (c) Cell adhesion on the peptide (0.1 mg/mL) modified substrate. (d) Cell adhesion on the peptide (0.5 mg/mL) modified substrate. Cells were stained with NucBlue and secondary antibody labeled with Alexa 488, and the acquired images were processed with ImageJ by subtracting background with the parameter "rolling ball radius of 50 pixels". Scale bars represent a distance of 500 μm.
